# Selective preservation of changes to standing balance control despite psychological and autonomic habituation to a postural threat

**DOI:** 10.1038/s41598-020-79417-5

**Published:** 2021-01-11

**Authors:** Martin Zaback, Minh John Luu, Allan L. Adkin, Mark G. Carpenter

**Affiliations:** 1 School of Kinesiology, University of British Columbia, 6108 Thunderbird Blvd., Vancouver, BC V6T 1Z4 Canada; 2 Department of Kinesiology, Brock University, St. Catharines, ON Canada; 3 Djavad Mowafaghian Centre for Brain Health, University of British Columbia, Vancouver, BC Canada; 4 International Collaboration on Repair Discoveries, University of British Columbia, Vancouver, BC Canada

**Keywords:** Emotion, Motor control

## Abstract

Humans exhibit changes in postural control when confronted with threats to stability. This study used a prolonged threat exposure protocol to manipulate emotional state within a threatening context to determine if any threat-induced standing behaviours are employed independent of emotional state. Retention of balance adaptations was also explored. Thirty-seven adults completed a series of 90-s standing trials at two surface heights (LOW: 0.8 m above ground, away from edge; HIGH: 3.2 m above ground, at edge) on two visits 2–4 weeks apart. Psychological and autonomic state was assessed using self-report and electrodermal measures. Balance control was assessed using centre of pressure (COP) and lower limb electromyographic recordings. Upon initial threat exposure, individuals leaned backward, reduced low-frequency centre of pressure (COP) power, and increased high-frequency COP power and plantar/dorsiflexor coactivation. Following repeated exposure, the psychological and autonomic response to threat was substantially reduced, yet only high-frequency COP power and plantar/dorsiflexor coactivation habituated. Upon re-exposure after 2–4 weeks, there was partial recovery of the emotional response to threat and few standing balance adaptations were retained. This study suggests that some threat-induced standing behaviours are coupled with the psychological and autonomic state changes induced by threat, while others may reflect context-appropriate adaptations resistant to habituation.

## Introduction

Fear of falling is prevalent amongst older adults^1^ and individuals with movement disorders^2,3^. Older adults with a self-reported fear of falling demonstrate differences in static^4^ and dynamic^5,6^ balance control compared to individuals matched for age and level of physical function. These observations suggest that emotional factors have the potential to directly influence the control of balance, potentially contributing to the increased risk of falling documented amongst individuals living with a fear of falling^7,8^. As a result, there is a clear need to understand the nature of fear-related balance changes and the potential for training to effectively impact these behaviours.

To examine how fear of falling influences balance control, different postural threat manipulations have been used. The underlying premise is that emotional and cognitive changes similar to those experienced on a daily basis by individuals living with a fear of falling can be induced by changing the perceived consequences or likelihood of falling^9–11^. Commonly used threat manipulations have involved either raising the height of the surface on which individuals stand (height-induced threat) or having individuals stand at ground level with or without the expectation of a sudden, unpredictable balance perturbation (threat of perturbation)^12^. With both threat manipulations, individuals demonstrate hallmarks of increased sympathetic activation (i.e., increased tonic electrodermal activity) and self-report greater anxiety and changes in cognitive appraisals that would be expected in individuals experiencing a fear of falling (i.e., reductions in balance-specific efficacy and perceived ability to cope with the threat)^11,13,14^. These changes in psychological and autonomic state are accompanied by changes in standing balance control that appear to be moderated by the context of the threat. With height-induced threat, individuals typically lean away from the platform edge and demonstrate higher frequency and smaller amplitude postural adjustments^12^. By contrast, with the threat of an unpredictable forward or backward perturbation, individuals lean forward and demonstrate higher frequency and larger amplitude postural adjustments^15,16^.

Recent work has shown that after being repeatedly exposed to height-induced threat^17^ or the threat of perturbation^18^, individuals’ emotional response to the threat was significantly attenuated, yet most changes in standing balance control were preserved. With repeated exposure to height, the only threat-induced changes in standing balance to show any attenuation included very high frequency centre of pressure (COP) power (> 1.8 Hz) and coactivation of plantar and dorsiflexor muscles^17^. By contrast, the posterior lean and reductions in COP amplitude and low-frequency COP power (< 0.05 Hz) did not habituate. Similar changes in COP dynamics were observed following repeated exposure to the threat of perturbation, with habituation limited to relatively high frequency COP oscillations (0.5–1.8 Hz) and mean power frequency (MPF) of COP^18^. It is unclear why this pattern of adaptation, or lack thereof, was observed. One possible explanation is that only some changes in standing balance control are related to the psychological and autonomic state changes induced by the threat. Unlike threat-induced changes in the average frequency content of COP oscillations, changes in leaning and COP amplitude have shown inconsistent correlations with self-report and physiological indicators of individuals’ emotional response to threat^13,14,17,19,20^ and exhibit different directions of change depending upon the type of threat experienced (height or perturbation threat)^12^. This could suggest that these changes in standing balance are employed in a context-dependent manner largely independent of individuals’ psychological and autonomic response to the threat.

Alternatively, there may be a non-linear relationship between some threat-induced changes in standing balance control and the magnitude of individuals’ emotional response to threat, such that complete, or near complete, attenuation of psychological and autonomic state changes induced by the threat are needed before specific standing balance adaptations begin to manifest. The aforementioned studies used relatively brief exposure periods, and self-reported and physiological measures of individuals’ emotional response to threat in these samples seldom approached values comparable to non-threatening conditions^17,18^. Thus, it remains possible that some threat-induced changes in standing balance control may begin to show adaptation only following a longer period of exposure that more thoroughly attenuates the cognitive-emotional responses to the threat.

Therefore, the current study used an extended exposure protocol to determine if threat-induced changes in standing balance persist following complete or near-complete adaptation of individuals’ psychological and autonomic response to a height-induced threat. To address this question, two separate sets of analyses were completed. The first set of analyses examined how threat-induced changes in standing balance adapt over the course of a long bout of repeated threat exposure across the sample as a whole. The second set of analyses focused exclusively on a subgroup of participants who demonstrated complete attenuation of their psychological and autonomic response to threat. It was expected that the majority of participants would show near complete adaptation of their psychological and autonomic response to threat and these changes would be accompanied by reductions in high-frequency COP power and plantar/dorsiflexor coactivation^17,21^. On the basis that threat-induced changes in leaning and COP amplitude are context-dependent and adopted largely independent of individuals’ psychological and autonomic response to threat, these were expected to show minimal change over the course of repeated threat exposure, even amongst individuals who showed complete adaptation of their psychological and autonomic response to threat.

A secondary aim of this study was to examine the extent to which standing balance adaptations acquired following repeated threat exposure are retained. Repeated exposure to activities perceived as threatening is an important component of cognitive-behavioural interventions designed to reduce fear of falling^22,23^. However, no studies to date have examined if any standing balance adaptations acquired during repeated threat exposure are retained when re-exposed to a similar threat. To address this question, participants from this study were re-exposed to the same height-induced postural threat 2–4 weeks after they completed the extended threat exposure protocol. Based on previous studies that have repeatedly exposed individuals to height-related situations, it was hypothesized that individuals’ psychological and autonomic response to threat would be attenuated (relative to their initial exposure to threat) following a short retention period^24,25^. This reduced emotional response to threat was expected to be accompanied by smaller threat-induced increases in high-frequency COP power and plantar/dorsiflexor coactivation. Other threat-induced changes in standing balance which were not expected to show adaptation with repeated threat exposure (i.e., leaning and COP amplitude and low frequency power) were not expected to be any different between visits.

## Methods

### Participants

Thirty-seven healthy young adults (mean age ± SD = 23.4 ± 4.4 years; 22 females) naïve to the postural threat manipulation participated in this study. Participants were free of musculoskeletal and neurological disorders that could impair their balance. No participants self-reported having an extreme fear of heights. All procedures were performed in accordance with relevant guidelines and regulations, and were reviewed and cleared by the University of British Columbia’s Clinical Research Ethics Board. Participants provided written informed consent.

Prior to completing the experimental procedures, participants provided basic demographic information and completed a battery of personality questionnaires. This included the trait version of the State-Trait Anxiety Inventory^26^ to assess trait anxiety, the Movement-Specific Reinvestment Scale^27^ to assess individuals’ propensity to reinvest attention in their movement, and the recreational subscale of the Domain Specific Risk-Taking Scale^28^ to assess physical risk-taking. Participant demographic and personality trait information are provided in Table 1.

**Table 1:** Demographic and personality information for participants and adaptor subgroup.

	Age	Sex	Height (cm)	Weight (kg)	STAI	MSRS	RT
Full sample	23.6 (4.6)	15M, 22F	171.9 (10.3)	66.2 (13.6)	39.7 (7.5)	36.7 (7.2)	24.8 (8.5)
Adaptors (n = 15)	21.8 (2.8)	4M, 11F	168.5 (10.7)	62.5 (13.1)	39.4 (6.8)	36.1 (8.0)	27.6 (7.3)

Values provided reflect means and standard deviations (with the exception of sex, in which case sums are provided). Scores range from 20–80 on the State-Trait Anxiety Inventory (STAI; higher scores reflect greater trait anxiety), 10–60 on the Movement-Specific Reinvestment Scale (MSRS; higher scores reflect greater propensity for movement reinvestment), and 6–42 on the Domain Specific Risk-Taking Scale (RT; higher scores reflect greater propensity for recreational risk-taking). Note, personality data were not collected for two participants, both of whom were in the Adaptor subgroup.

### Procedures

Participants completed a series of 90-s quiet standing trials at two different postural threat conditions on two visits to the lab separated by approximately 2–4 weeks (mean retention period ± SD = 20.3 ± 4.0 days). Throughout each trial, participants stood barefoot on a force plate (BP400600, AMTI, USA) positioned at the edge of a hydraulic lift (1.52 m × 2.13 m; Pentalift, Canada) with a stance width equal to the length of their foot and their toes were aligned to the anterior edge of the force plate. The borders of participants’ feet were traced onto the force plate to ensure consistent foot placement across all trials within each session. At the LOW condition, the platform was positioned at its lowest height (0.8 m above ground) and a table (0.6 m × 1.6 m) was positioned in front of the platform, creating 60 cm of continuous support surface in front of the participant^29^. For the HIGH condition, the table was removed, and the platform was elevated 3.2 m above the ground.

During their first visit (initial session), participants completed 2 quiet standing trials at the LOW condition before (LOW_pre_) and after (LOW_post_) a block of 20 trials at the HIGH condition. This blocked presentation order was selected to maximize within-session habituation of the psychological and autonomic response to threat^25,30^. Throughout all trials, participants were instructed to stand quietly with their arms at their sides and fixate on an eye-level visual target positioned 3.8 m in front of them. To minimize potential fatigue effects associated with the extensive number of standing trials, at least 2-min of seated rest were completed between all trials. Prior to the experimental trials, participants completed a 90-s practice trial at the LOW condition to minimize potential first trial effects^9^.

**Figure 1:**
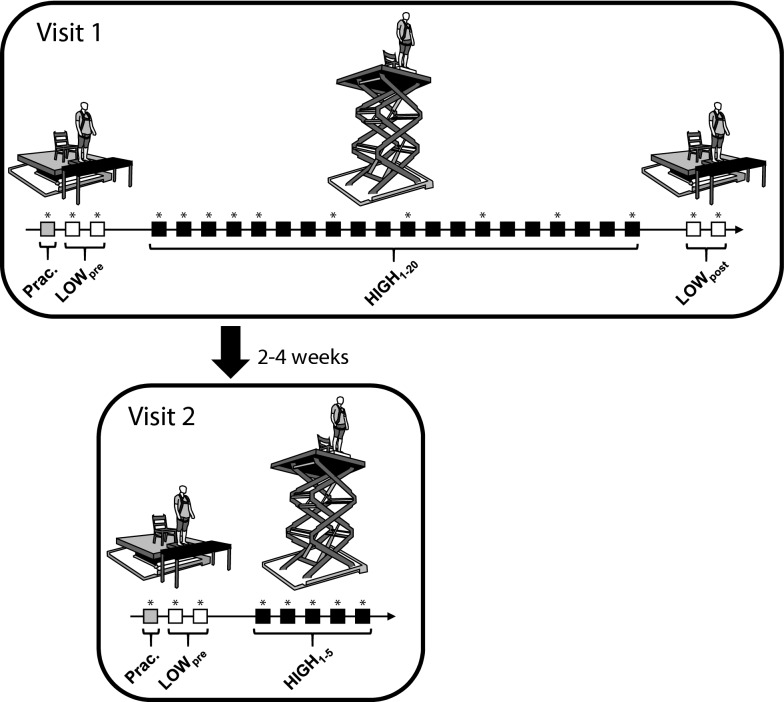
Flow chart outlining the experimental protocol. Participants completed a series of 90-s trials of quiet standing at LOW (0.8 m above ground, away from edge) and HIGH (3.2 m above ground, at edge) threat conditions on two visits to the lab separated by 2–4 weeks. Each square represents a single 90-s trial; open squares represent trials completed at the LOW condition, while filled black squares represent trials completed at the HIGH condition. During both visits, participants first completed a single 90-s practice trial (grey square) at the LOW condition. Trials in which questionnaires were administered to assess cognitive and emotional state are indicated with an asterisk.

During their second visit (retention session), participants completed an abbreviated version of the initial session. In particular, they completed two standing trials at the LOW condition (LOW_pre_) followed by 5 trials at the HIGH condition. Similar to the initial session, the standing trials were 90-s in length and at least 2-min of seated rest were completed between consecutive trials. Participants also completed a 90-s practice trial prior to the LOW_pre_ trials. No participants reported engaging in any height-related activities during the time between the two visits. Figure 1 provides a schematic illustration of experimental procedures across both visits.

Throughout all trials during both visits, participants wore a safety harness that was securely fastened by climbing rope to an overhead support beam. The tension of the rope was adjusted such that the harness did not provide support that would assist in the balance task, but would support the participant’s body weight above the platform surface in the event of a fall. A trained spotter was also seated behind the participants to prevent a fall if the participant appeared unsteady. No falls occurred during this experiment and the spotter did not have to intervene to provide support at any point.

### Data collection

Ground reaction forces and moments were sampled from the force plate at 100 Hz. Electromyography (EMG) was recorded bilaterally from the soleus (SOL) and tibialis anterior (TA) using Ag/AgCl surface electrodes positioned in a bipolar arrangement (interelectrode distance of ~ 1.25 cm). EMG data were sampled at 3000 Hz (Telemyo 2400R G2, Noraxon, USA), bandpass filtered online (10–1000 Hz), and then A-D sampled at 2000 Hz (Power1401, CED, UK). Skin conductance was recorded from Ag/AgCl surface electrodes placed on the thenar and hypothenar eminences of the non-dominant hand and sampled at 100 Hz (Model 2502SA, CED, UK).

Self-reports of emotional and cognitive state were probed at pre-defined trials throughout the experiment. During the initial session, this included all trials at the LOW condition and trials 1–5, 8, 11, 14, 17, and 20 at the HIGH condition. This order was selected since the greatest reduction in the emotional response to threat is likely to occur within the first five trials at the HIGH threat condition^17^. Self-reports were probed at all trials during the retention session (Fig. 1). Before each of these trials, participants reported how confident they were that they could maintain their balance and avoid a fall during the upcoming balance task. After these trials, they completed single-item questions that assessed cognitive and somatic anxiety and fear of falling. Responses to each of these questions were completed on visual analog scales (VAS) ranging from 0 to 100. Higher scores reflected greater balance confidence, cognitive and somatic anxiety, and fear of falling. Scores on the measures of cognitive and somatic anxiety were averaged to create a state anxiety score^17,18^. Focus of attention was also assessed after these trials using a 5-item questionnaire that asked participants to rate how much they thought about or paid attention to different information. Single questions were used to probe attention toward (1) movement processes (i.e., conscious control or monitoring of movement); (2) threat-related stimuli (i.e., feelings of anxiety or worry); (3) self-regulatory strategies (i.e., coping strategies to help remain calm, confident, and/or focused); (4) task objectives (i.e., constraints of the task); and (5) task-irrelevant information (i.e., thoughts unrelated to the balance task). Responses were rated on 9-point Likert scales, with higher scores indicating greater attention to each loci of attention^17,18^. The items and terminology incorporated into this questionnaire were derived from qualitative research investigating changes in attention associated with height-induced threat^31^.

### Data analysis

#### Centre of pressure (COP) outcomes

Ground reaction forces and moments were low-pass filtered offline (10 Hz cutoff, 2nd order dual-pass Butterworth filter). From these data, only anterior–posterior COP was calculated since the effect of height-induced threat is greatest in this plane when facing the platform edge^12^. From the COP data, the mean COP position (MPOS-COP) was calculated to provide a measure of how far individuals leaned away from the platform edge. The mean was then subtracted from the COP signal to remove the bias and a linear detrend was applied. From these data, root mean square (RMS-COP) and mean power frequency (MPF-COP) were calculated to provide estimates of the amplitude and average frequency content of COP oscillations, respectively. When individuals stand under conditions of height-induced threat, MPF-COP typically increases, and recent work has shown this is due to reductions in the amplitude of lower frequency COP oscillations (< 0.05 Hz) and increases in the amplitude of higher frequency COP oscillations (> 0.5 Hz)^17^. Furthermore, following a relatively short period of repeated threat exposure, only oscillations greater than 1.8 Hz show significant attenuation^17^. Thus, mean COP power was calculated within three frequency bands: 0–0.05 Hz (low), 0.5–1.8 Hz (medium), and 1.8–5.0 Hz (high)^17,18^.

#### Tonic muscle activity and coactivation

EMG data from bilateral SOL and TA were debiased, full-wave rectified, and normalized to mean rectified EMG during maximal voluntary contractions. From these data, tonic activation levels were calculated as mean rectified EMG (averaged between both legs). EMG data were then low-pass filtered (3 Hz cutoff, 5th order dual-pass Butterworth filter) creating linear envelopes. SOL-TA coactivation for each leg was then determined by calculating the absolute overlap of SOL and TA linear envelopes^32,33^. Coactivation estimates for both legs were then averaged for each participant.

#### Sympathetic arousal

To provide an estimate of sympathetic arousal, non-specific electrodermal response frequency (EDR.freq) was calculated from skin conductance data. Skin conductance data were first low-pass filtered (1 Hz cutoff, 5th order dual-pass Butterworth filter) and then a customized algorithm identified and counted all electrodermal responses with peak amplitudes greater than 0.05 µS^34^. All identified EDRs were visually inspected and any false positive due to movement artifacts were rejected^35^.

### Identification of “Adaptors”

Participants’ whose emotional response to threat returned to LOW threat values during the first visit were identified as “Adaptors”. To be considered an Adaptor, the following criteria needed to be met. First, participants needed to have a self-reported increase of at least 5 points on the fear of falling VAS during the first HIGH trial relative to the mean of the first two LOW trials. Participants then needed measures of self-reported fear of falling and EDR.freq to return to LOW threat values. A threshold for what would be considered LOW threat values for each participant was calculated as the mean + 1SD for each of these measures based on the first two LOW trials. The HIGH trial at which point both fear of falling and EDR measures returned to a value equal to or less than the LOW threshold was noted and all psychological, autonomic, and standing balance outcomes for this trial and the next HIGH trial were calculated and averaged (HIGH_adapted_). The only exception to this was if these criteria were met only by the time of the final HIGH trial, in which case there would be no subsequent HIGH trial, so only data from HIGH_20_ were used (n = 1). The HIGH_adapted_ trials were included in the statistical procedures as opposed to the last two HIGH trials (i.e., HIGH_19 and 20_) in order to minimize possible fatigue or boredom effects which may manifest with continued exposure after the point of complete adaptation.

### Statistics

#### Within-session adaptation

To examine how measures of psychological and autonomic state and standing balance adapt over the course of repeated threat exposure, a series of two-way (Threat: LOW vs. HIGH; Trial: Pre vs. Post) repeated measures ANOVAs were conducted. For the factor Trial, data were averaged across the first two (LOW_pre_) and last two (LOW_post_) LOW trials for the Pre and Post levels of the LOW condition, while the first (HIGH_1_) and last (HIGH_20_) HIGH trials were used for the Pre and Post levels of the HIGH condition, respectively. Significant threat × trial interactions were followed-up with Bonferroni corrected paired-samples t-tests that examined the effect of threat across both levels of trial and the effect of trial across both levels of threat (alpha = 0.0125).

A separate set of statistics were conducted for individuals identified as Adaptors in order to determine if threat-induced changes in standing balance control are preserved in individuals whose emotional response to threat returned fully to LOW threat values. Dependent measures for the following trials were included in one-way repeated measures ANOVAs: LOW_pre_ (average of the first two LOW trials), HIGH_pre_ (average of first two HIGH trials), and HIGH_adapted_. The reason blocks of two trials were averaged was to reduce variability of the standing balance measures, since fewer participants were expected to be included in these analyses. Significant main effects were followed up with Bonferroni corrected paired-samples t-tests comparing HIGH_pre_ and HIGH_adapted_ with LOW_pre_ (alpha = 0.025).

#### Retention of adaptations

To determine if any adaptations were retained between visits, two-way (Visit: 1 vs. 2; Trial: Δ HIGH_1_ vs. Δ HIGH_5_) repeated measures ANOVAs were conducted for measures of psychological and autonomic state and standing balance control for all participants. For the factor Trial, dependent measures at HIGH_1_ and HIGH_5_ were expressed as change scores relative to LOW_pre_ data from respective visits (Δ HIGH_1_ = HIGH_1_ – LOW_pre_; Δ HIGH_5_ = HIGH_5_ – LOW_pre_). Significant interactions were followed up with Bonferroni corrected paired-samples t-tests examining the effect of trial across both levels of visit and the effect of visit across both levels of trial (alpha = 0.0125).

## Results

Two participants (1 female) reported increases in fear of falling from the first to last HIGH trial during the initial session. Because the primary aim of this study was to determine if threat-induced changes in standing balance persist after *attenuation* of the emotional response to threat, these participants were excluded from statistical analyses. Due to technical issues, EMG data were missing for one participant and skin conductance data were missing for another. Several dependent measures were positively skewed; this included low-, medium-, and high-frequency COP power, tonic TA activation, and SOL-TA coactivation. To correct for this non-normality, log transformations were applied to these data.

### Visit 1 within-session adaptation: psychological and autonomic outcomes

**Figure 2:**
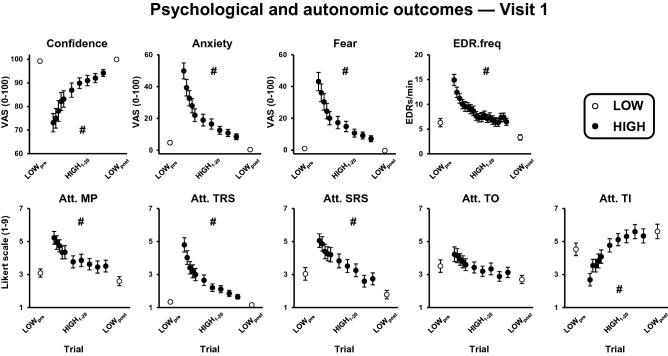
Effect of threat and trial on psychological and autonomic outcomes. Data presented reflect group means and standard errors. The first two and last two LOW trials have been averaged (LOW_pre_ and LOW_post_, respectively) and are represented as open circles, while data across HIGH trials 1–20 are represented as filled black circles. Note, self-reported measures at the HIGH condition were only collected on trials 1-5, 8, 11, 14, 17, and 20 as illustrated. Att. = attention toward; EDR.freq = non-specific electrodermal response frequency; MP = movement process; SRS = self-regulatory strategies; TI = task-irrelevant information; TO = task objectives; TRS = Threat-related stimuli; VAS = visual analog scale. Hashtags indicate significant threat × trial interactions resulting from a reduced effect of threat as a function of repeated exposure.

Significant threat × trial interactions were observed for all psychological and autonomic state measures except for attention toward task objectives (p-values ≤ 0.017; Table 2). Follow-up comparisons (summarized in Table 3) revealed that when individuals were initially exposed to the threat, they were less confident and more anxious, fearful, and physiologically aroused. Participants also directed more attention toward their movement, threat-related stimuli, and self-regulatory strategies, and less attention toward task-irrelevant information when initially exposed to the threat. For each of the aforementioned outcomes, the effect of threat was significantly reduced after repeated threat exposure. In all cases, the reduced effect of threat resulted from each psychological and autonomic state measure showing a rapid, non-linear attenuation across the block of HIGH trials (Fig. 2). While the effect of threat was substantially reduced, all outcomes, except attention toward task-irrelevant information, remained significantly different from LOW_post_ values (Table 3). For attention toward task objectives (which did not show a significant threat × trial interaction), there were significant main effects of threat and trial, such that individuals directed more attention toward task objectives in the HIGH condition independent of trial, but less attention toward this information across both threat conditions over time (Fig. 2; Table 2). Collectively, this pattern of results indicates that repeated exposure to the HIGH condition resulted in substantial within-session habituation of individuals’ psychological and autonomic response to threat.

**Table 2:** Two-way repeated-measures ANOVAs (threat: LOW vs. HIGH; trial: pre vs. post) examining within-session habituation of self-report, autonomic, and standing balance outcomes at visit 1.

	Threat × trial interaction			Threat main effect			Trial main effect		
	F	*p*	η_p_^2^	F	*p*	η_p_^2^	F	*p*	η_p_^2^
**Psychological and autonomic outcomes**									
Confidence	36.306	< **0.001**	0.516	45.032	** < 0.001**	0.570	40.141	** < 0.001**	0.541
Anxiety	69.469	< **0.001**	0.671	85.034	** < 0.001**	0.714	89.698	** < 0.001**	0.725
Fear of falling	51.102	< **0.001**	0.600	55.222	** < 0.001**	0.619	51.577	** < 0.001**	0.603
EDR.freq	32.727	< **0.001**	0.498	101.787	** < 0.001**	0.755	94.735	** < 0.001**	0.742
Att. MP	18.284	< **0.001**	0.350	41.194	** < 0.001**	0.548	27.369	** < 0.001**	0.446
Att. TRS	58.237	< **0.001**	0.631	65.455	** < 0.001**	0.658	71.278	** < 0.001**	0.677
Att. SRS	6.262	**0.017**	0.156	46.394	** < 0.001**	0.577	46.693	** < 0.001**	0.579
Att. TO	0.558	0.460	0.016	10.274	**0.003**	0.232	9.985	**0.003**	0.227
Att. TI	12.970	**0.001**	0.276	18.436	** < 0.001**	0.352	21.354	** < 0.001**	0.386
**Standing balance outcomes**									
MPOS-COP	0.085	0.772	0.003	60.840	** < 0.001**	0.642	7.938	**0.008**	0.189
RMS-COP	6.004	**0.020**	0.150	12.505	**0.001**	0.269	0.017	0.898	< 0.001
MPF-COP	5.530	**0.025**	0.140	32.120	** < 0.001**	0.486	8.529	**0.006**	0.201
LF-COP power	3.026	0.091	0.082	13.388	**0.001**	0.283	0.158	0.694	0.005
MF-COP power	5.904	**0.021**	0.148	18.883	** < 0.001**	0.357	12.759	**0.001**	0.273
HF-COP power	11.347	**0.002**	0.250	38.360	** < 0.001**	0.530	14.319	** < 0.001**	0.296
Tonic SOL activation	8.219	**0.007**	0.199	8.593	**0.006**	0.207	0.049	0.826	0.001
Tonic TA activation	16.234	** < 0.001**	0.330	65.618	** < 0.001**	0.665	22.730	** < 0.001**	0.408
SOL-TA Co-Ac	25.387	** < 0.001**	0.435	66.157	** < 0.001**	0.667	29.053	** < 0.001**	0.468

EDR.freq: non-specific electrodermal response frequency; Att.: attention toward; MP: movement processes; TRS: threat-related stimuli; SRS: self-regulatory strategies; TO: task objectives; TI: task-irrelevant information; MPOS: mean position; RMS: root mean square; MPF: mean power frequency; LF: low-frequency (0–0.05 Hz); MF: medium-frequency (0.5–1.8 Hz); HF: high-frequency (1.8–5 Hz); SOL: soleus; TA: tibialis anterior; Co-Ac: coactivation. Significant p-values (<0.05) are boldfaced.

**Table 3:** Follow-up pairwise comparisons for significant threat × trial interactions for two-way RM-ANOVAs examining within-session habituation at visit 1.

	HIGH_1_ vs. LOW_pre_		HIGH_1_ vs. HIGH_20_		HIGH_20_ vs. LOW_post_		LOW_pre_ vs. LOW_post_	
	Δ (± SD)	p-value	Δ (± SD)	p-value	Δ (± SD)	p-value	Δ (± SD)	p-value
**Psychological and autonomic outcomes**								
Confidence	**− 26.10 (± 22.78)**	** < 0.001**	**− 21.11 (± 20.14)**	** < 0.001**	**− 5.74 (± 8.65)**	** < 0.001**	**− 0.76 (± 1.58)**	**0.008**
Anxiety	**44.92 (± 28.53)**	** < 0.001**	**41.19 (± 26.64)**	** < 0.001**	**8.03 (± 10.30)**	** < 0.001**	**4.29 (± 6.06)**	** < 0.001**
Fear	**41.90 (± 32.49)**	** < 0.001**	**35.80 (± 29.41)**	** < 0.001**	**7.49 (± 11.09)**	** < 0.001**	1.39 (± 3.16)	0.014
EDR.freq	**8.63 (± 5.43)**	** < 0.001**	**8.48 (± 4.78)**	** < 0.001**	**3.14 (± 3.06)**	** < 0.001**	**2.97 (± 4.03)**	** < 0.001**
Att. MP	**2.14 (± 1.90)**	** < 0.001**	**1.71 (± 1.79)**	** < 0.001**	**0.91 (± 1.33)**	** < 0.001**	0.49 (± 1.15)	0.016
Att. TRS	**3.46 (± 2.50)**	** < 0.001**	**3.14 (± 2.26)**	** < 0.001**	**0.50 (0.74)**	** < 0.001**	0.19 (0.47)	0.026
Att. SRS	**2.01 (± 2.24)**	** < 0.001**	**2.31 (± 2.45)**	** < 0.001**	**0.96 (± 1.20)**	** < 0.001**	**1.26 (± 1.38)**	** < 0.001**
Att. TI	**− 1.84 (± 2.37)**	** < 0.001**	**− 2.66 (± 2.94)**	** < 0.001**	− 0.271 (± 1.40)	0.260	− 1.09 (± 2.48)	0.014
**Standing balance outcomes**								
RMS-COP (mm)	− 0.226 (± 1.00)	0.191	0.279 (± 1.087)	0.138	− **0.748 (± 1.058)**	** < 0.001**	− 0.243 (± 0.997)	0.159
MPF-COP (Hz)	**0.162 (± 0.197)**	** < 0.001**	**0.086 (± 0.180)**	**0.008**	**0.096 (± 0.105)**	** < 0.001**	0.020 (± 0.068)	0.089
MF-COP power (mm^2^/Hz)	**0.379 (± 0.633)**	** < 0.001**	**0.295 (± 0.623)**	**0.002**	**0.119 (± 0.250)**	**0.005**	0.036 (0.138)	0.099
HF-COP power (mm^2^/Hz)	**0.059 (0.117)**	** < 0.001**	**0.049 (± 0.115)**	** < 0.001**	**0.012 (± 0.020)**	** < 0.001**	0.001 (0.005)	0.255
Tonic SOL activation (% MVC)	− 0.369 (± 2.762)	0.441	0.566 (± 2.588)	0.211	− **1.628 (± 1.881)**	** < 0.001**	− **0.692 (± 1.455)**	**0.009**
Tonic TA activation (% MVC)	**5.792 (± 4.838)**	** < 0.001**	**3.388 (± 4.183)**	** < 0.001**	**2.723 (± 3.280)**	** < 0.001**	0.319 (± 0.982)	0.073
SOL-TA Co-Ac (% MVC)	**2.487 (± 2.041)**	** < 0.001**	**1.724 (± 2.000)**	** < 0.001**	**0.924 (± 1.328)**	** < 0.001**	0.161 (± 0.531)	0.057

Mean differences (Δ) and p-values for each comparison are presented. EDR.freq: non-specific electrodermal response frequency; Att.: attention toward; MP: movement processes; TRS: threat-related stimuli; SRS: self-regulatory strategies; TO: task objectives; TI: task-irrelevant information; COP: centre of pressure; RMS: root mean square; MPF: mean power frequency; LF: low-frequency (0–0.05 Hz); MF: medium-frequency (0.5–1.8 Hz); HF: high-frequency (1.8–5 Hz); MVC: maximal voluntary contraction; SOL: soleus; TA: tibialis anterior; Co-Ac: coactivation. Significant comparisons after Bonferroni correction (alpha 0.0125) are boldfaced.

### Visit 1 within-session adaptation: standing balance outcomes

**Figure 3:**
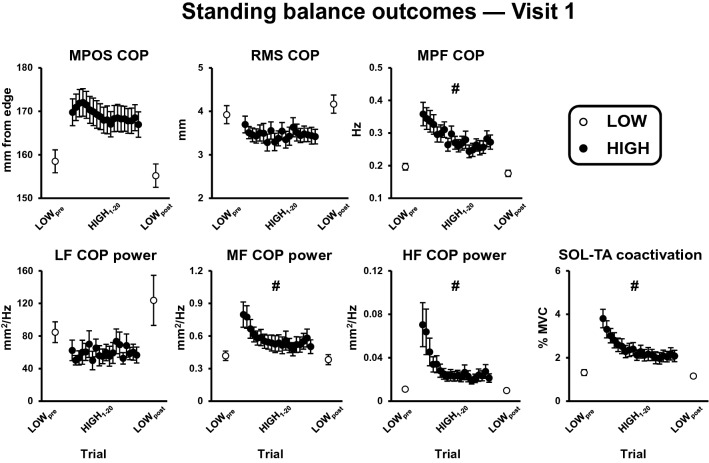
Effect of threat and trial on standing balance outcomes. Data presented reflect group means and standard errors. The first two and last two LOW trials have been averaged (LOW_pre_ and LOW_post_, respectively) and are represented as open circles, while data across HIGH trials 1–20 are represented as filled black circles. COP = centre of pressure; HF = high frequency (1.8–5 Hz); LF = low frequency (0–0.05 Hz); MF = medium frequency (0.5–1.8 Hz); MPF = mean power frequency; MPOS = mean position; RMS = root mean square; SOL-TA = soleus-tibialis anterior. Hashtags indicate significant threat × trial interactions resulting from a reduced effect of threat as a function of repeated exposure.

Significant threat × trial interactions were observed for MPF-COP, medium- (0.5–1.8 Hz) and high- (1.8–5 Hz) frequency COP power, tonic TA activation, and SOL-TA coactivation (p-value range: 0.001 to 0.025; Table 2). Follow-up comparisons (summarized in Table 3) revealed that when individuals were initially exposed to the threat, they demonstrated greater MPF-COP, medium- and high-frequency COP power, tonic TA activation, and SOL-TA coactivation. By the last HIGH trial, the effect of threat was attenuated for each of these outcomes but remained significantly different from LOW_post_ values. The reduced effect of threat resulted from significant reductions in each standing balance outcome occurring across only the HIGH condition (Table 3). Similar to the psychological and autonomic state measures, these standing balance outcomes demonstrated rapid, non-linear attenuation over the course of repeated threat exposure (Fig. 3).

**Figure 4:**
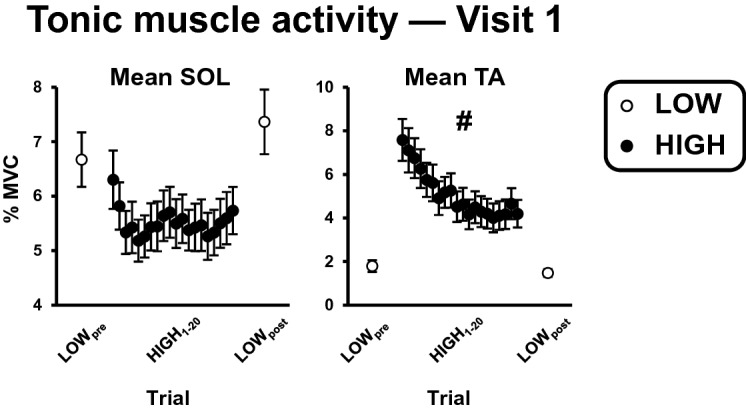
Effect of threat and trial on tonic activation of soleus (SOL) and tibialis anterior (TA) muscles. Data presented reflect group means and standard errors. The first two and last two LOW trials have been averaged (LOW_pre_ and LOW_post_, respectively) and are represented as open circles, while data across HIGH trials 1–20 are represented as filled black circles. Hashtags indicate significant threat × trial interactions resulting from a reduced effect of threat as a function of repeated exposure.

Significant threat × trial interactions were also observed for RMS-COP and tonic SOL activation (p-values ≤ 0.020; Table 1). Unlike the aforementioned standing balance outcomes, follow-up comparisons revealed that these interactions resulted from the effect of threat only becoming significant after the initial exposure to height (Table 3; Figs. 3, 4). When individuals were first exposed to the threat, RMS-COP and tonic SOL activation were lower, but did not significantly differ from LOW_pre_ values (p-values: 0.191 and 0.441, respectively). However, by the last HIGH trial both outcomes were significantly lower compared to LOW_post_ (p-values < 0.001). For tonic SOL activation, this was the result of significant decreases in SOL activation occurring from the first to last HIGH trial, while for RMS-COP, this was the result of non-significant increases from the first to last LOW trials and non-significant decreases from the first to last HIGH trial (Table 3). This pattern of results indicates that the effect of threat for each of these outcomes does not diminish over the course of repeated threat exposure, but instead becomes more pronounced.

For MPOS-COP and low-frequency COP power (0–0.05 Hz), there were no significant threat × trial interactions. However, there were significant main effects of threat for each of these outcomes (p-values < 0.001), such that individuals reduced the power of low-frequency COP oscillations and leaned away from the platform edge at the HIGH compared to LOW condition independent of trial. There was also a significant main effect of trial for MPOS-COP (*p* = 0.008), such that individuals leaned forward as a function of time independent of threat condition (Table 2; Fig. 3). Collectively, this indicates that the effect of threat for these outcomes does not diminish over the course of repeated threat exposure despite substantial reductions in the psychological and autonomic response to threat.

### Visit 1 within-session adaptation for the “Adaptor” subgroup: psychological and autonomic outcomes

Fifteen participants (demographics summarized in Table 1) met the criteria to be identified as “Adaptors” (median trial at which point criteria to be fully adapted were met = HIGH_8_; range: HIGH_3_–HIGH_20_).

**Figure 5:**
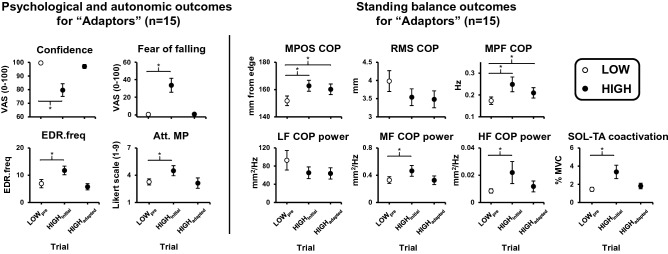
Effect of threat and trial on psychological, autonomic, and standing balance outcomes in the “Adaptor” subgroup (n = 15). Data presented reflect group means and standard errors. The first two LOW trials for each participant were averaged (LOW_pre_) and are presented as open circles. The first two HIGH trials for each participant were averaged (HIGH_initial_). The trial at which point the participant was identified as an “Adaptor” and the next HIGH trial were averaged (HIGH_adapted_). Both HIGH_initial_ and HIGH_adapted_ are presented as filled black circles. Att. MP = attention toward movement processes; COP = centre of pressure; EDR.freq = non-specific electrodermal response frequency; HF = high frequency (1.8–5 Hz); LF = low frequency (0–0.05 Hz); MF = medium frequency (0.5–1.8 Hz); MPF = mean power frequency; MPOS = mean position; RMS = root mean square; SOL-TA = soleus-tibialis anterior; VAS = visual analog scale. Brackets with asterisks indicate significant planned follow-up comparisons (p < 0.025).

One-way repeated measures ANOVAs were significant for all psychological and autonomic outcomes (p-value range: < 0.001 to 0.041; Table 4). Follow-up planned comparisons demonstrated a significant effect of threat when individuals were first exposed to the HIGH condition (HIGH_initial_ vs. LOW_pre_) for all outcomes except for attention toward task objectives and task-irrelevant information. In particular, when initially exposed to threat, the “Adaptors” were less confident and more anxious, fearful, and physiologically aroused. In addition, these participants directed more attention toward movement processes, threat-related stimuli, and self-regulatory strategies. However, by the trial at which point they met the criteria for complete adaptation, the effect of threat for each of these outcomes was no longer significant (HIGH_adapted_ vs. LOW_pre_) (p-value range: 0.094 to 1.00; Fig. 5).

**Table 4:** One-way repeated measures ANOVAs for “Adaptor” subgroup.

	F	*p*	η_p_^2^
**Psychological and autonomic outcomes**			
Confidence	19.829	** < 0.001**	0.586
Anxiety	25.918	** < 0.001**	0.649
Fear of falling	18.729	**0.001**	0.572
EDR.freq	26.348	** < 0.001**	0.653
Att. MP	3.807	**0.034**	0.214
Att. TRS	18.124	** < 0.001**	0.564
Att. SRS	5.533	**0.009**	0.283
Att. TO	7.599	**0.007**	0.352
Att. TI	9.969	**0.001**	0.416
**Standing balance outcomes**			
MPOS-COP	12.499	** < 0.001**	0.606
RMS-COP	2.716	0.084	0.162
MPF-COP	6.138	**0.014**	0.305
LF-COP power	1.808	0.183	0.114
MF-COP power	8.014	**0.002**	0.364
HF-COP power	7.363	**0.008**	0.345
Tonic SOL activation	2.948	0.070	0.185
Tonic TA activation	14.082	** < 0.001**	0.520
SOL-TA Co-Ac	15.853	** < 0.001**	0.549

EDR.freq: non-specific electrodermal response frequency; Att.: attention toward; MP: movement processes; TRS: threat-related stimuli; SRS: self-regulatory strategies; TO: task objectives; TI: task-irrelevant information; MPOS: mean position; RMS: root mean square; MPF: mean power frequency; LF: low-frequency (0–0.05 Hz); MF: medium-frequency (0.5–1.8 Hz); HF: high-frequency (1.8–5 Hz); SOL: soleus; TA: tibialis anterior; Co-Ac: coactivation. Significant p-values (<0.05) are boldfaced.

### Visit 1 within-session adaptation for the “Adaptor” subgroup—standing balance outcomes

Significant one-way repeated measures ANOVAs were observed for MPOS-COP, MPF-COP, medium- and high-frequency COP power, tonic TA activation, and SOL-TA coactivation (p-value range: < 0.001 to 0.008; Table 5). For each of these outcomes, follow-up planned comparisons demonstrated a significant effect of threat when individuals were first exposed to the HIGH condition. For medium- and high-frequency COP power, tonic TA activation, and SOL-TA coactivation, the effect of threat was no longer significant once participants demonstrated complete adaptation of their psychological and autonomic response to threat (p-value range: 0.279 to 0.751; Fig. 5). However, the effect of threat remained significant for MPOS-COP (*p* < 0.001) and MPF-COP (*p* = 0.017). One-way repeated measures ANOVAs did not reach significance for RMS-COP (*p* = 0.084), low-frequency COP power (*p* = 0.183), and tonic SOL activation (*p* = 0.07).

**Table 5:** Two-way repeated-measures ANOVAs (visit: 1 vs. 2; trial: Δ HIGH_1_ vs. Δ HIGH_5_) examining retention and within-session adaptation of self-report, autonomic, and standing balance outcomes across visits.

	Visit × trial interaction			Visit main effect			Trial main effect		
	F	*p*	η_p_^2^	F	*p*	η_p_^2^	F	*p*	η_p_^2^
**Psychological and autonomic outcomes**									
Confidence	0.063	0.803	0.002	25.204	** < 0.001**	0.441	23.69	** < 0.001**	0.425
Anxiety	1.756	0.194	0.052	29.968	** < 0.001**	0.484	67.160	** < 0.001**	0.677
Fear of falling	0.064	0.802	0.002	23.918	** < 0.001**	0.428	43.606	** < 0.001**	0.577
EDR.freq	0.026	0.872	0.001	3.479	0.072	0.101	75.863	** < 0.001**	0.710
Att. MP	0.212	0.648	0.007	4.176	**0.050**	0.119	16.041	** < 0.001**	0.341
Att. TRS	0.070	0.793	0.002	17.685	** < 0.001**	0.356	48.802	** < 0.001**	0.604
Att. SRS	0.254	0.618	0.008	0.714	0.404	0.022	15.258	** < 0.001**	0.323
Att. TO	0.021	0.885	0.001	0.772	0.386	0.024	15.201	** < 0.001**	0.322
Att. TI	0.721	0.402	0.022	2.218	0.146	0.065	35.645	** < 0.001**	0.527
**Standing balance outcomes**									
MPOS-COP	6.371	**0.017**	0.166	1.442	0.239	0.043	0.383	0.540	0.012
RMS-COP	2.226	0.145	0.065	< 0.001	0.992	< 0.001	0.492	0.488	0.015
MPF-COP	0.099	0.755	0.003	2.140	0.153	0.063	11.771	**0.002**	0.269
LF-COP power	0.578	0.453	0.018	0.009	0.925	< 0.001	0.350	0.559	0.011
MF-COP power	0.202	0.656	0.006	4.016	0.054	0.112	7.376	**0.011**	0.187
HF-COP power	0.460	0.503	0.014	8.129	**0.008**	0.203	21.418	** < 0.001**	0.401
Tonic SOL activation	7.747	**0.009**	0.195	0.631	0.433	0.019	8.230	**0.007**	0.205
Tonic TA activation	1.541	0.224	0.046	3.257	0.081	0.092	37.602	** < 0.001**	0.540
SOL-TA Co-Ac	0.085	0.773	0.003	2.488	0.125	0.072	59.271	** < 0.001**	0.649

EDR.freq: non-specific electrodermal response frequency; Att.: attention toward; MP: movement processes; TRS: threat-related stimuli; SRS: self-regulatory strategies; TO: task objectives; TI: task-irrelevant information; MPOS: mean position; RMS: root mean square; MPF: mean power frequency; LF: low-frequency (0–0.05 Hz); MF: medium-frequency (0.5–1.8 Hz); HF: high-frequency (1.8–5 Hz); SOL: soleus; TA: tibialis anterior; Co-Ac: coactivation. Significant p-values (<0.05) are boldfaced.

### Visit 1 and 2 between-session comparisons: psychological and autonomic outcomes

Two participants did not return for visit 2, reducing the sample of participants to 33 for these analyses.

**Figure 6:**
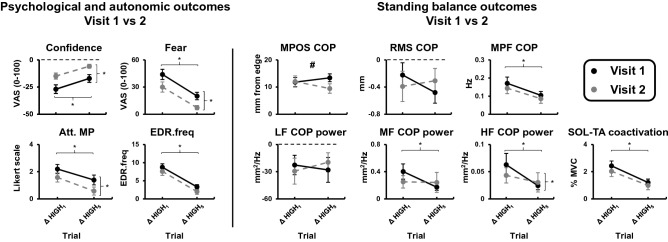
Effect of visit and trial on psychological, autonomic, and standing balance outcomes. Data presented reflect group means and standard errors of change scores (Δ HIGH_1_ = HIGH_1_ – LOW_pre_; Δ HIGH_5_ = HIGH_5_ – LOW_pre_). Change scores for visit 1 and 2 are presented as filled black and grey circles, respectively. Att. MP = attention toward movement processes; COP = centre of pressure; EDR.freq = non-specific electrodermal response frequency; HF = high frequency; LF = low frequency; MF = medium frequency; MPF = mean power frequency; MPOS = mean position; RMS = root mean square; SOL = soleus; TA = tibialis anterior; VAS = visual analog scale. Vertical and horizontal brackets with asterisks indicate significant main effect of visit and trial, respectively (*p* < 0.05). Hashtags represent significant visit × trial interaction (*p* < 0.05).

No significant visit × trial interactions were observed for any psychological and autonomic state outcomes. However, significant main effects of trial were observed for all psychological and autonomic outcomes (all p-values < 0.001; Table 5) such that individuals’ emotional response to threat was attenuated from HIGH_1_ to HIGH_5_ independent of the visit (Fig. 6; Table 5). Significant main effects of visit were observed for most self-report outcomes. In particular, the changes observed between HIGH and LOW threat were smaller on the retention compared to initial session for confidence, anxiety, fear of falling, and attention toward movement and threat-related stimuli. No main effect of visit was observed for EDR.freq (*p* = 0.072) and attention toward self-regulatory strategies (*p* = 0.404), task objectives (*p* = 0.386), and task-irrelevant information (*p* = 0.146) (Table 5).

### Visit 1 and 2 between-session comparisons—standing balance outcomes

Significant visit × trial interactions were observed for MPOS-COP and tonic SOL activation. For MPOS-COP, this interaction resulted from individuals leaning non-significantly further backward from the HIGH_1_ to HIGH_5_ during the initial session (*p* = 0.210) and non-significantly further forward during the retention session (*p* = 0.021; Fig. 6). For tonic SOL activation, the interaction resulted from SOL activity showing further decreases from HIGH_1_ to HIGH_5_ during the initial session (*p* = 0.001), but not changing across these same trials during the retention session (*p* = 0.513; Fig. 6). Of the remaining variables, significant main effects of trial were observed for MPF-COP, medium- and high-frequency COP power, tonic TA activation, and SOL-TA co-activation. In all cases, threat-induced changes in these standing balance outcomes were attenuated from HIGH_1_ to HIGH_5_ independent of visit (p-value range: < 0.001 to 0.011; Table 5). A significant main effect of visit was only observed for high-frequency COP power, with individuals demonstrating smaller threat-induced increases in high-frequency COP power during the retention session (*p* = 0.008; Table 5). No significant interactions or main effects were observed for RMS-COP or low-frequency COP power (Table 5).

## Discussion

The primary aim of this study was to determine if threat-induced changes in standing balance persist following near complete attenuation of individuals’ psychological and autonomic response to a height-induced threat. Consistent with previous work, when individuals were initially exposed to the threat, they leaned significantly away from the platform edge, significantly increased MPF-COP and plantar/dorsiflexor coactivation, and tended to decrease RMS-COP^12^. Changes in MPF-COP were the result of significant reductions in low-frequency COP power (< 0.05 Hz) and increases in higher-frequency COP power (> 0.5 Hz). With repeated threat exposure, individuals demonstrated a rapid and substantial attenuation of their psychological and autonomic response to the threat. However, only some threat-induced changes in standing balance tended to follow a similar pattern of adaptation. In particular, MPF-COP, higher-frequency COP power (> 0.5 Hz), and plantar/dorsiflexor coactivation were significantly reduced over the course of repeated threat exposure. Other threat-induced changes in standing balance, including the posterior lean and decreased amplitude of COP displacements and low-frequency COP power, did not change over the course of repeated threat exposure. This same pattern of adaptation was still observed in a subgroup of participants whose psychological and autonomic response to threat returned to LOW threat values. These observations suggest that while some threat-induced changes in standing balance vary closely with individuals’ psychological and autonomic response to threat, other balance changes may be employed in a given threat context independent of psychological and autonomic state.

A secondary aim of this study was to determine if standing balance adaptations are retained across repeated visits. Consistent with our hypotheses, individuals self-reported a reduced psychological response to threat upon re-exposure following a 2–4-week retention period. However, individuals’ autonomic response to threat, as measured by the frequency of non-specific electrodermal responses, did not differ significantly across visits. Furthermore, few standing balance outcomes showed retention, with only high-frequency COP power (> 1.8 Hz) demonstrating a reduced effect of threat at the time of the retention session. These results suggest that some standing balance adaptations acquired during a single block of repeated threat exposure can be retained, but retention of additional balance adaptations may require that the psychological, along with the autonomic response to threat, are minimized upon re-exposure.

### Standing balance outcomes prone to adaptation following repeated threat exposure

Previous studies have shown that threat-induced changes in MPF-COP, high-frequency COP oscillations, and plantar/dorsiflexor coactivation are highly correlated with individuals’ psychological and autonomic response to threat^17,21^. Thus, it is not surprising that these changes in standing balance control appeared to change in parallel with measures of psychological and autonomic state over the course of repeated threat exposure and even returned to LOW threat values in the Adaptor subgroup. Collectively, this further supports the notion that these particular changes in standing balance are heavily dependent on individuals’ psychological and autonomic response to threat.

It is unclear if these particular threat-induced changes in standing balance serve a functional role when individuals are confronted with a threat to balance. Plantar/dorsiflexor coactivation increases ankle joint stiffness during standing, but there is little evidence this provides additional stability^36,37^. Rather, plantar/dorsiflexor coactivation is an energetically costly compensatory strategy^38^ that may interfere with voluntary and reactive postural control^39,40^. Some have suggested that increased ankle stiffness may improve postural recovery following perturbations, but only if accompanied by reductions in sensorimotor gain^41^. Moreover, some perturbations, such as support surface rotations, are more destabilizing as ankle stiffness and stretch sensitivity increase^42,43^. Since postural threat has been shown to facilitate muscle afferent^11,44,45^ and vestibular reflex gain^46,47^, such changes in coactivation may be maladaptive in this context. High-frequency COP oscillations may also serve a limited functional role, since COP movement greater than 2 Hz has a negligible influence on the control of sway during standing due to the body’s large moment of inertia^48^. If anything, these unnecessary movements may interfere with or mask balance-relevant somatosensory inputs^49,50^. Thus, it would appear favourable that these particular components of the behavioural response to threat are most prone to rapid habituation with repeated threat exposure.

### Context-dependent standing balance changes

Previous studies have shown that when individuals are repeatedly exposed to height or the threat of an impending perturbation, some threat-induced changes in standing balance control are largely invariant^17,18^. This has been taken to suggest that some components of the behavioural response to threat may be adopted irrespective of the psychological and autonomic state changes induced by the threat. The present study supports this supposition and casts doubt on the possibility that the lack of standing balance adaptations seen in previous studies were the result of insufficient habituation^17,18^. This is because threat-induced changes in COP mean position and low-frequency power still showed little sign of attenuation, even in the Adaptor subgroup. Furthermore, threat-induced reductions in RMS-COP and tonic SOL activation tended to become greater over the course of repeated threat-exposure.

While these standing balance outcomes do not appear to be strongly influenced by individuals’ psychological and autonomic response to threat, previous studies suggest they are heavily dependent on the nature of the postural threat. Both the threat of height and impending perturbation elicit similar changes in psychological and autonomic state^11^. However, when standing with the expectation of an unpredictable forward or backward perturbation, individuals lean forward and increase their amplitude of postural sway^15,16,51^. This is the opposite to what is observed with a height-induced threat, where individuals tend to lean away from the edge of the platform and restrict their postural sway^12^. In both instances, these standing balance changes appear appropriate given the nature of the threat. Leaning backward and limiting postural sway reduces the likelihood of a fall over the platform edge when standing at height, while leaning forward and increasing postural sway may facilitate compensatory stepping and postural recovery when standing on the ground and responding to a perturbation^51,52^. Thus, these components of the behavioural response to threat appear to reflect context-appropriate adaptations to minimize the likelihood of a fall or injury in the face of unique postural challenges.

### Retention of psychological, autonomic, and standing balance outcomes

When participants were re-exposed to the height-induced threat 2–4 weeks after their initial block of exposure, they demonstrated within-session adaptations similar to what was seen during their initial session. In particular, their self-reported and autonomic response to threat decreased over time, and this was accompanied by similar reductions in MPF-COP, higher-frequency COP power (> 0.5 Hz), and plantar/dorsiflexor coactivation. One exception was mean position of COP, as individuals showed a tendency to lean slightly forward over the course of the abbreviated exposure period during the retention session only.

One of the most striking findings was that few of the standing balance adaptations acquired during the initial visit were retained, with only high-frequency COP power (> 1.8 Hz) showing a smaller effect of threat at follow-up. This was likely a consequence of only modestly retained reductions in the psychological and autonomic response to threat. While individuals did report greater confidence and less fear, anxiety, and attention toward their movement when they were re-exposed to the threat after 2–4 weeks, their autonomic response, as measured by the frequency of non-specific electrodermal responses, was not significantly reduced. Spontaneous recovery of a previously habituated emotional response is commonly observed following exposure therapy^53^, and the exposure protocol used in the present study was not well-suited to minimize this^54–56^.

The present study used a massed and constant exposure protocol. This form of exposure was selected in order to maximize within-session habituation of the psychological and autonomic response to threat^25,30^. This was crucial to address the primary goal of this study, which was to determine if threat-induced changes in standing balance persist after near complete attenuation of the psychological and autonomic response to threat. Although effective in this regard, this form of exposure does not promote the encoding, consolidation, and retrieval processes thought to be critical for long-term fear reduction^54–56^. Furthermore, the exposure protocol was entirely unguided. That is, participants were not instructed to avoid using coping strategies, such as distraction or cognitive avoidance, which can facilitate within-session habituation, but limit long-term fear reduction^55,57^. Exposure protocols which explicitly limit the use of such strategies and vary the conditions of the exposure in terms of timing, intensity, and/or the context of threat presentations are more effective in minimizing individuals’ emotional response to a similar threat on subsequent exposures^30,54,55,57,58^. However, these types of exposure protocols were considered undesirable for the present study since they tend to result in sustained within-session anxiety and arousal^54,55^. Nevertheless, given that the standing balance adaptations acquired over the course of repeated threat exposure generally appear beneficial to the control of balance, future work should determine if improved retention can be obtained using different exposure protocols.

### Limitations, implications, and future directions

A strength of this study was that the extended threat exposure protocol was sufficient to completely diminish the psychological and autonomic response to threat to LOW values in a subgroup of participants during the initial session. However, since only 15 participants met the criteria to be included in this subgroup, results related to these analyses are underpowered and should be interpreted with caution. Multiple factors may have contributed to between-subjects variability in habituation to the height-induced threat. Personality traits that have been shown to predict individuals’ response to height-induced postural threat^59^ were recorded and did not differ between Adaptors and Non-adaptors (Table 1). However, it is unclear how other personality traits or previous experiences with height-related activities may have influenced habituation in this context. The results of this study are also only generalizable to healthy young adults. Previous work has shown that young and older adults show a similar pattern of psychological, autonomic, and standing balance adaptations when repeatedly exposed to the threat of perturbation^18^. However, it is not clear if this is the case for different types of postural threat or with different clinical populations (e.g., Parkinson’s disease, stroke survivors, etc.). We believe it is important to first investigate the underlying mechanisms of emotional influences on balance in healthy individuals unbiased from any underlying fear or anxiety issues known to accompany balance disorders due to age or disease^1–3^. However, given the therapeutic potential of repeated threat exposure for minimizing fear of falling and fall risk in different clinical populations, future work is needed to determine if similar psychological, autonomic, and standing balance adaptations are observed in different clinical populations in response to unique threat scenarios.

This study also demonstrated that the emotional response to threat decreased rapidly with repeated exposure, such that the effect of threat for most psychological and autonomic outcomes was reduced by more than 50% within as few as five 90-s standing trials. Studies using postural threat manipulations need to take this rapid habituation into account, particularly if multiple conditions or trials need to be completed under a HIGH threat scenario. Since changes in the autonomic and behavioural response to threat are poorly retained across visits, studies requiring participants to be highly fearful or aroused across multiple threat conditions could be advised to complete their experiment over separate days to minimize confounds related to habituation.

## Conclusions

This study demonstrates that while some threat-induced changes in standing balance are heavily dependent on individuals’ psychological and autonomic response to threat, and are therefore amendable to intervention, others are more resistant to change and appear to be employed in a primarily context-dependent manner. While the changes to standing balance observed following a single block of repeated threat exposure appear functional to the control of balance, they are not readily retained after 2–4 weeks without exposure. This is likely a consequence of inadequate emotional learning inherent to the massed and constant exposure protocol used in the present study. However, given the potential for exposure interventions to minimize potentially maladaptive changes in balance, it is incumbent upon future work to determine how to most effectively structure exposure exercises to maximize long-term fear reductions and associated balance changes. Such interventions may have the potential to reduce fall-risk amongst individuals living and/or working with a fear of falling.

## Supplementary Information

Supplementary Information.

## Data Availability

All data generated and analyzed during this study are included in this published article (and its Supplementary information file).

## References

[1] Legters K (2002). Fear of falling. Phys. Ther..

[2] Adkin AL, Frank JS, Jog MS (2003). Fear of falling and postural control in Parkinson’s disease. Mov. Disord..

[3] Schmid AA (2015). Fear of falling in people with chronic stroke. Am. J. Occup. Ther..

[4] Maki BE, Holliday PJ, Topper AK (1991). Fear of falling and postural performance in the elderly. J. Gerontol..

[5] Okada S, Hirakawa K, Takada Y, Kinoshita H (2001). Relationship between fear of falling and balancing ability during abrupt deceleration in aged women having similar habitual physical activities. Eur. J. Appl. Physiol..

[6] Uemura K (2012). Fear of falling is associated with prolonged anticipatory postural adjustment during gait initiation under dual-task conditions in older adults. Gait Posture.

[7] Friedman SM, Munoz B, West SK, Rubin GS, Fried LP (2002). Falls and fear of falling: Which comes first? A longitudinal prediction model suggests strategies for primary and secondary prevention. J. Am. Geriatr. Soc..

[8] Li F, Fisher KJ, Harmer P, McAuley E, Wilson NL (2003). Fear of falling in elderly persons: Association with falls, functional ability, and quality of life. J. Gerontol. B-Psychol..

[9] Adkin AL, Frank JS, Carpenter MG, Peysar GW (2000). Postural control is scaled to level of postural threat. Gait Posture.

[10] Brown LA, Frank JS (1997). Postural compensations to the potential consequences of instability: Kinematics. Gait Posture.

[11] Horslen BC, Murnaghan CD, Inglis JT, Chua R, Carpenter MG (2013). Effects of postural threat on spinal stretch reflexes: Evidence for increased muscle spindle sensitivity?. J. Neurophysiol..

[12] Adkin AL, Carpenter MG (2018). New insights on emotional contributions to human postural control. Front. Neurol..

[13] Carpenter MG, Adkin AL, Brawley LR, Frank JS (2006). Postural, physiological and psychological reactions to challenging balance: Does age make a difference?. Age Ageing.

[14] Hauck LJ, Carpenter MG, Frank JS (2008). Task-specific measures of balance efficacy, anxiety, and stability and their relationship to clinical balance performance. Gait Posture.

[15] Johnson KJ, Zaback M, Tokuno CD, Carpenter MG, Adkin AL (2019). Exploring the relationship between threat-related changes in anxiety, attention focus, and postural control. Psychol. Res..

[16] Shaw JA, Stefanyk LE, Frank JS, Jog MS, Adkin AL (2012). Effects of age and pathology on stance modifications in response to increased postural threat. Gait Posture.

[17] Zaback M, Adkin AL, Carpenter MG (2019). Adaptation of emotional state and standing balance parameters following repeated exposure to height-induced postural threat. Sci. Rep..

[18] Johnson KJ, Zaback M, Tokuno CD, Carpenter MG, Adkin AL (2019). Repeated exposure to the threat of perturbation induces emotional, cognitive, and postural adaptations in young and older adults. Exp. Gerontol..

[19] Davis JR, Campbell AD, Adkin AL, Carpenter MG (2009). The relationship between fear of falling and human postural control. Gait Posture.

[20] Huffman JL, Horslen BC, Carpenter MG, Adkin AL (2009). Does increased postural threat lead to more conscious control of posture?. Gait Posture.

[21] Wuehr M (2019). Fear of heights in virtual reality saturates 20 to 40 m above ground. J. Neurol..

[22] Zijlstra GA (2009). Effects of a multicomponent cognitive behavioral group intervention on fear of falling and activity avoidance in community-dwelling older adults: Results of a randomized controlled trial. J. Am. Geriatr. Soc..

[23] Dorresteijn TAC (2016). Effectiveness of a home-based cognitive behavioral program to manage concerns about falls in community-dwelling, frail older people: Results of a randomized controlled trial. BMC Geriatr..

[24] Baker A (2010). Does habituation matter? Emotional processing theory and exposure therapy for acrophobia. Behav. Res. Ther..

[25] Lang AJ, Craske MG (2000). Manipulation of exposure-based therapy to reduce return of fear: A replication. Behav. Res. Ther..

[26] Spielberger CD, Gorsuch RL, Lushene R, Vagg PR, Jacobs GA (1983). Manual for the State-Trait Anxiety Inventory.

[27] Masters, R. S., Eves, F. F., & Maxwell, J. P. Development of a movement-specific reinvestment scale. In: ISSP 11th World Congress of Sport Psychology (2005).

[28] Blais A-R, Weber EU (2006). A Domain-Specific Risk-Taking (DOSPERT) scale for adult populations. Judgm. Decis. Mak..

[29] Carpenter MG, Frank JS, Silcher CP, Peysar GW (2001). The influence of postural threat on the control of upright stance. Exp. Brain. Res..

[30] Rowe MK, Craske MG (1998). Effects of varied-stimulus exposure training on fear reduction and return of fear. Behav. Res. Ther..

[31] Zaback M, Carpenter MG, Adkin AL (2016). Threat-induced changes in attention during tests of static and anticipatory postural control. Gait Posture.

[32] Crenna P, Inverno M, Frigo C, Palmieri R, Fedrizzi E (1992). Pathophysiological profile of gait in children with cerebral-palsy. Med. Sport Sci..

[33] Frost G, Dowling J, Dyson K, Bar-Or O (1997). Cocontraction in three age groups of children during treadmill locomotion. J. Electromyogr. Kines..

[34] Boucsein W (2012). Publication recommendations for electrodermal measurements. Psychophysiol..

[35] Boucsein W (2012). Electrodermal Activity.

[36] Latash ML (2018). Muscle coactivation: Definitions, mechanisms, and functions. J. Neurophysiol..

[37] Yamagata M, Falaki A, Latash ML (2019). Effects of voluntary agonist-antagonist coactivation on stability of vertical posture. Mot. Control.

[38] Peterson DS, Martin PE (2010). Effects of age and walking speed on coactivation and cost of walking in healthy adults. Gait Posture.

[39] Nagai K (2013). Effects of muscle coactivation during quiet standing on dynamic postural control in older adults. Arch. Gerontol. Geriatr..

[40] Nagai K, Okita Y, Ogaya S, Tsuboyama T (2017). Effect of higher muscle coactivation on standing postural response to perturbation in older adults. Aging Clin. Exp. Res..

[41] Le Mouel C, Brette R (2019). Anticipatory coadaptation of ankle stiffness and sensorimotor gain for standing balance. PLos Comput. Biol..

[42] Allum JHJ, Carpenter MG, Honegger F, Adkin AL, Bloem BR (2002). Age-dependent variations in the directional sensitivity of balance corrections and compensatory arm movements in man. J. Physiol..

[43] Carpenter MG, Allum JH, Honegger F (1999). Directional sensitivity of stretch reflexes and balance corrections for normal subjects in the roll and pitch planes. Exp. Brain Res..

[44] Davis JR (2011). Human proprioceptive adaptations during states of height-induced fear and anxiety. J. Neurophysiol..

[45] Horslen BC, Zaback M, Inglis JT, Blouin JS, Carpenter MG (2018). Increased human stretch reflex dynamic sensitivity with height-induced postural threat. J. Physiol..

[46] Horslen BC, Dakin CJ, Inglis JT, Blouin JS, Carpenter MG (2014). Modulation of human vestibular reflexes with increased postural threat. J. Physiol..

[47] Naranjo EN, Allum JH, Inglis JT, Carpenter MG (2015). Increased gain of vestibulospinal potentials evoked in neck and leg muscles when standing under height-induced postural threat. Neuroscience.

[48] Gage WH, Winter DA, Frank JS, Adkin AL (2004). Kinematic and kinetic validity of the inverted pendulum model in quiet standing. Gait Posture.

[49] Collins JJ, Imhoff TT, Grigg P (1997). Noise-mediated enhancements and decrements in human tactile sensation. Phys. Rev. E.

[50] Mildren RL, Bent LR (2016). Vibrotactile stimulation of fast-adapting cutaneous afferents from the foot modulates proprioception at the ankle joint. J. Appl. Physiol..

[51] Maki BE, Whitelaw RS (1993). Influence of expectation and arousal on center-of-pressure responses to transient postural perturbations. J. Vestib. Res..

[52] Rajachandrakumar R, Mann J, Schinkel-Ivy A, Mansfield A (2018). Exploring the relationship between stability and variability of the centre of mass and centre of pressure. Gait Posture.

[53] Rachman S (1989). The return of fear: Review and prospect. Clin. Psychol. Rev.

[54] Craske MG (2008). Optimizing inhibitory learning during exposure therapy. Behav. Res. Ther..

[55] Craske MG, Treanor M, Conway CC, Zbozinek T, Vervliet B (2014). Maximizing exposure therapy: An inhibitory learning approach. Behav. Res. Ther..

[56] Lang AJ, Craske MG, Bjork RA (1999). Implications of a new theory of disuse for the treatment of emotional disorders. Clin. Psychol..

[57] Foa EB, Huppert JD, Cahill SP, Rothbaum BO (2006). Emotional processing theory: An update. Pathological Anxiety: Emotional Processing in Etiology and Treatment.

[58] Tsao JCI, Craske MG (2000). Timing of treatment and return of fear: Effects of massed, uniform-, and expanding-spaced exposure schedules. Behav. Ther..

[59] Zaback M, Cleworth TW, Carpenter MG, Adkin AL (2015). Personality traits and individual differences predict threat-induced changes in postural control. Hum. Mov. Sci..

